# Phylogenetic Relationships of Three *Ramaria* Species Based on Mitochondrial Genome Analysis

**DOI:** 10.1002/ece3.70901

**Published:** 2025-02-12

**Authors:** Xianyi Wang, Zhongyao Guo, Jiawei Tao, Gongyou Zhang, Guoyu Wang, Yaping Wang, Yaohang Long, Hongmei Liu

**Affiliations:** ^1^ Engineering Research Center of Health Medicine Biotechnology of Institution of Higher Education of Guizhou Province Guiyang China; ^2^ Engineering Research Center of Medical Biotechnology, School of Biology and Engineering Guizhou Medical University Guiyang China; ^3^ Key Laboratory of Biology and Medical Engineering, Immune Cells and Antibody Engineering Research Center of Guizhou Province, School of Biology and Engineering Guizhou Medical University Guiyang China; ^4^ The High Efficacy Application of Natural Medicinal Resources Engineering Center of Guizhou Province (The Key Laboratory of Optimal Utilization of Natural Medicine Resources), School of Pharmaceutical Sciences Guizhou Medical University Guiyang China; ^5^ School of Public Health, the Key Laboratory of Environmental Pollution Monitoring and Disease Control, Ministry of Education Guizhou Medical University Guiyang China; ^6^ State Key Laboratory of Functions and Applications of Medicinal Plants Guizhou Medical University Guiyang China

**Keywords:** clavarioid fungi, evolution, mitochondrial genome, phylogenetic analysis, *Ramaria* species

## Abstract

*Ramaria* has been a remarkable genus throughout the history of macrofungi. However, there is a lack of information on this genus of macrofungi. This study determined the order of nucleotides in the mitochondrial genomes (mitogenomes) of three *Ramaria* species, followed by a detailed investigation of the obtained genetic information. Circular mitogenomes of *Ramaria brunnecliacina*, *R*. *ichnusensis*, and *R. flavescens* had sizes of 78,960, 61,851, and 81,282 bp, respectively. The genomes exhibited variations in genetic content, gene length, tRNA, and codon usage. *Ramaria* mitogenomes demonstrated variable evolutionary rates across several protein‐coding genes. The results revealed significant gene rearrangements in *Ramaria* mitogenomes, including gene displacement and tRNA duplication. Utilizing Bayesian inference and maximum likelihood methods on a comprehensive set of conserved mitochondrial proteins, we generated a well‐supported phylogenetic tree for Basidiomycota. This analysis revealed that *R*. *brunneciacina* and *R. flavescens* are closely related, while confirming the paraphyletic nature of the *Ramaria* genus and its genetic affinity with other species of the subclass Phallomycetidae. This study presents a basic structure for understanding the evolutionary dynamics, genetic makeup, and taxonomy categorization of this significant fungal community.

## Graphical abstract

This study sequenced and analyzed the mitochondrial genomes of *Ramaria brunnecliacina*, *R. ichnusensis*, and *R. flavescens* to investigate their genetic characteristics and phylogenetic relationships. The genomes revealed significant variations in gene content, structure, and evolutionary rates, with notable gene rearrangements. Phylogenetic analysis provided a clearer understanding of the evolutionary position of Ramaria within the Basidiomycota, showing close genetic relationships with species in the subclass Phallomycetidae.

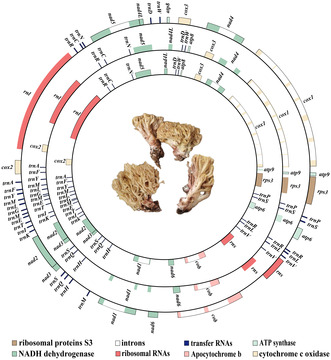

## 1 Introduction

The colorful and densely branched sporangia of *Ramaria* species are frequently referred to as coral fungi. These fungi are major ectomycorrhizal associates of conifers. Regarding the toxicity of *Ramaria*, certain fungi, including *Ramaria flavobrunnescens* and *R. rufescens* , are classified as toxic (Huang et al. 2009; Perez‐Moreno and Ferrera‐Cerrato 1995; Scheid et al. 2022), whereas several studies have identified certain substances as nontoxic (Aprotosoaie et al. 2017; Khaund and Joshi 2014; Liu et al. 2014, 2015). Polysaccharides, sesquiterpenes, and glucans extracted from *Ramaria* possess antioxidant, antigenotoxic, antitumor, antimicrobial, and immune‐enhancing properties (Barros et al. 2008; Bhanja et al. 2013; Dong, Hou, and Ding 2020; Fu et al. 2022). *Ramaria* species are widely distributed throughout southwestern China, encompassing Yunnan, Sichuan, Tibet, and Guizhou. These fungi are symbionts and decomposers and hence are crucial to the health of forests and the cycling of nutrients in forest ecosystems (Zhang, Yang, and Ge 2005; Petersen 1987, 1988; Petersen and Zang 1986). Ultimately, the precise identification and categorization of *Raymaria* species are essential for their effective utilization and exploitation.

*Ramaria* species belonging to the order Gomphales and the subclass Phallomycetidae, occupy a unique position in the evolutionary history of higher basidiomycetes. Traditionally, *Ramaria* has relied on morphological traits for identification. However, distinguishing ectomycorrhizae based solely on morphological criteria is challenging due to their striking similarity (Nouhra et al. 2005). Reliance on physical traits often leads to misclassification, hindering the identification and utilization of *Ramaria* species. To address these challenges, scientists combined morphological and molecular data, including nuclear and mitochondrial DNA sequences, to elucidate the evolutionary relationships among Geastrales, Gomphales, Hysterangiales, and Phallales (Giachini et al. 2010). Investigating the evolutionary connections and differences among species morphologically akin to *Ramaria*, particularly those within the Clavariaceae family, is crucial (Stielow et al. 2015). Despite sharing physical similarities, some species may have significant genetic variations (Humpert et al. 2001). Grasping these evolutionary dynamics will improve the comprehension of their biological interactions and better their taxonomy categorization.

The mitochondrial genome, often termed the mitogenome, is a critical component in studying the evolutionary dynamics of Basidiomycetes. Thought to have been derived from bacterial ancestors through endosymbiosis, the mitogenome, sometimes referred to as the “second genome” of eukaryotes (Gray, Burger, and Lang 2001), possesses unique properties that include its exclusive maternal inheritance, independent origin, and a variety of molecular markers. These characteristics make it an indispensable tool for tracing phylogenetic relationships across species (Abuduaini et al. 2021; Basse 2010). The arrangement of mitochondrial genes, changes in conserved gene sequences, the shifting landscape of introns, variations in tRNA structure, and diverse codon usage patterns all provide a deep reservoir of genetic data, vital for understanding species evolution and adaptability (Fonseca et al. 2021; Ren, Zhang, and Zhang 2021; Wu et al. 2021; Zhang, Bai, et al. 2021). Despite its significance, research into fungal mitogenomes lags behind that of animals, where mitochondrial mutations are often linked to diseases and have proven instrumental in understanding systematics and population dynamics (Du, Yu, and Yan 2022; Pickett et al. 2022; Zhong, Madry, and Cucchiarini 2022). In fungi, however, the connections between mitogenome variations and critical factors such as growth, development, and stress response are still largely unexplored (Garcia‐Souto et al. 2022; Wolfsberger et al. 2022; Zhang, Yang, et al. 2021; Araujo et al. 2021). Sequencing complete fungal mitogenomes aims to uncover how mutations in mitochondrial DNA affect fungal biology and traits. The studies have highlighted that even closely related fungal species can show substantial differences in gene order, repeat sequence frequency, gene quantity, and intron presence (Fonseca et al. 2021; Li et al. 2021; Férandon et al. 2010). Although a typical set of protein‐coding genes (PCGs) such as *atp6*, *atp8*, and *cox1* is usually found in Basidiomycetes, the pronounced variability in fungal mitogenome architecture complicates assembling a full genomic sequence. Currently, fewer than 1000 mitogenomes from basidiomycete species have been identified, a small fraction compared with the vast number of species described in nature.

The scarcity of available mitogenomes has hindered our ability to fully comprehend the genetic diversity and evolutionary processes within Basidiomycota (Sandor, Zhang, and Xu 2018). Leveraging mitogenomic information offers significant benefits, such as enhanced precision in deciphering evolutionary lineages and detecting species that are difficult to identify through traditional means (Li and Lu 2024). The traditional morphological methods have proven inadequate for accurately classifying Gomphales, underscoring the critical need to address this limitation (Nilsen et al. 2024). Through the study of mitogenomes, researchers have uncovered deeper phylogenetic relationships and gained fresh insights into the evolutionary trajectories of these fungi. Recent studies have highlighted the necessity of integrating molecular and morphological data to develop a more holistic approach to fungal taxonomy (Tao et al. 2024). Combining mitogenomic data with traditional morphological analysis presents a more resilient framework for classifying species, particularly within the genus *Ramaria*. This integrative approach is crucial for discovering previously unknown species and refining our understanding of evolutionary relationships within Gomphales and its related orders. Additionally, advancements in next‐generation sequencing (NGS) have greatly enhanced our capability to sequence and analyze mitogenomes, enabling a level of taxonomic precision that was previously unattainable. As a result, the use of mitochondrial DNA is increasingly recognized in the scientific literature as an effective tool for resolving intricate classification challenges in Basidiomycetes.

The primary objective of this research was to assemble and annotate the mitochondrial genomes of three *Ramaria* species, including *R*. *brunnecliacina*, *R*. *ichnusensis*, and *R. flavescens* , successively by conducting a comparative analysis with the mitochondrial genomes of other members of the subclass Phallomycetidae. The objective of the study was to detailedly analyze and describe the *Ramaria* mitogenomes, compare them to other species in the Phallomycetidae group, and determine the position of *Ramaria* within the Basidiomycota phylum by examining a combination of mitochondrial genes.

## 2 Materials and Methods

### 2.1 Sample Acquisition and Preservation

Three *Ramaria* specimens were gathered in August 2023 in Niubang Township, Yulong Township, Weining County, Bijie City, Guizhou Province. Sterile instruments were utilized to gather specimens designated by strain numbers YL2, LY5, and LY6, which were immediately stored in sterile, sealed bags to avert external contamination. To ensure preservation, the samples were stored at −20°C to maintain DNA integrity for prolonged use, whereas a portion of the specimens was air‐dried for subsequent morphological analysis. Subsequent to morphological identification, the specimens were conveyed to the laboratory of Guizhou Medical University for further processing.

To reduce the risk of surface contamination, the outside of each specimen was sterilized with 75% ethanol, followed by multiple rinses in sterile distilled water to ensure the complete removal of disinfectant residues. DNA was extracted from fresh or frozen tissue for molecular analysis utilizing a modified CTAB method (Doyle and Doyle 1987). The specimens were first homogenized in liquid nitrogen to rupture the cellular structure, thereafter undergoing DNA extraction using CTAB buffer. A further purification process removed potential inhibitors to guarantee DNA purity. The DNA quality was assessed using agarose gel electrophoresis, and its concentration was quantified with a spectrophotometer to verify its suitability for further sequencing. All samples were preserved at −20°C during the whole process until needed for further analysis.

### 2.2 Mitogenome Assembly and Annotation

To sequence the mitogenomes in this research, NGS was used, and the sequences were subsequently assembled using Geneious Prime 2023.2.1. The genomes that were put together were subsequently matched up with the mitochondrial reference sequence of *R. rubella* (NC 068232) to order to make comparisons. The PCGs, open reading frames (ORFs), rRNAs, tRNAs, and introns in the three *Ramaria* mitogenomes were annotated using the MFannot (Valach et al. 2014) and MITOS (Bernt et al. 2013) tools. Both methods utilize mitochondrial genetic code 4. ORFs (Coordinators NR 2017) that exceed 100 amino acids were subjected to additional refining or annotation using the NCBI Open Reading Frame Finder (Bleasby and Wootton 1990). Subsequently, BLASTP searches were conducted against the NCBI nonredundant protein database. To guarantee precision, the tRNA genes that were previously annotated were subjected to a thorough verification using tRNAscan‐SE v2.0.12 (Lowe and Chan 2016). Ultimately, the three *Ramaria* mitochondrial genomes were generated by using OGDraw v1.3.1 (Lohse et al. 2013) to create physical maps.

### 2.3 Analysis of Mitochondrial Genome Sequences

For the analysis of base compositions in *Ramaria* and other Phallomycetidae mitochondrial genomes, researchers utilized DNASTAR Lasergene v7.1 (http://www.dnastar.com/). Strand asymmetry was assessed by calculating GC skew ([G − C]/[G + C]) and AT skew ([A − T]/[A + T]) using conventional methods. The Sequence Manipulation Suite was applied to investigate codon usage within the *Ramaria* mitogenome, adhering to mitochondrial genetic code 4. To evaluate the substitution rates of nonsynonymous (Ka) and synonymous (Ks) mutations in the PCGs across *Ramaria* and Phallomycetidae species, DnaSP v6.10.01 (Rozas et al. 2017) was used. Subsequently, the genetic distances among the 15 PCGs were calculated using the Kimura‐2‐parameter (K2P) model through MEGA v11.0.10 (Caspermeyer 2016).

### 2.4 Phylogenetic Analysis

We used a comprehensive molecular approach incorporating mitochondrial genes to establish a robust and accurate phylogenetic framework for Basidiomycota. We created two distinct datasets based on fifteen conserved PCGs and the ribosomal large (*rnl*) and small (*rns*) subunit genes from 77 species within Basidiomycota. The first dataset, PCG, comprised the concatenated sequences of the fifteen conserved PCGs. The second dataset, PCG12, included these genes' combined first and second codon positions. Our primary focus was to elucidate the phylogenetic relationships among the species *R. brunnecliacina*, *R. ichnusensis*, and *R. flavescens* . The goal was to use mitochondrial DNA to build a detailed phylogenetic tree that accurately positions these species within the broader evolutionary context of Basidiomycota. For this study, *Dematophora necatrix* and *Xylaria hypoxylon* from the phylum Ascomycota served as outgroups. Mitochondrial genes were individually aligned using the MAFFT v7.490 (Katoh, Rozewicki, and Yamada 2019) program, after which the protein sequences were combined into unified datasets through PhyloSuite v1.2.2. software (Zhang et al. 2020). A partition homogeneity test was performed to assess potential conflicts in phylogenetic signals across different genes. The optimal evolutionary models and partitioning strategies for the mitochondrial gene dataset were identified using PartitionFinder 2.1.1 (Lanfear et al. 2017). Phylogenetic trees were then constructed using both Bayesian inference (BI) and maximum likelihood (ML) methods, with BI analysis executed in MrBayes v3.2.6 (Ronquist et al. 2012) and ML analysis conducted via IQ‐tree v1.6.3 (Nguyen et al. 2015).

## 3 Results

### 3.1 Characterization of the Three *Ramaria* Mitogenomes

**FIGURE 1:**
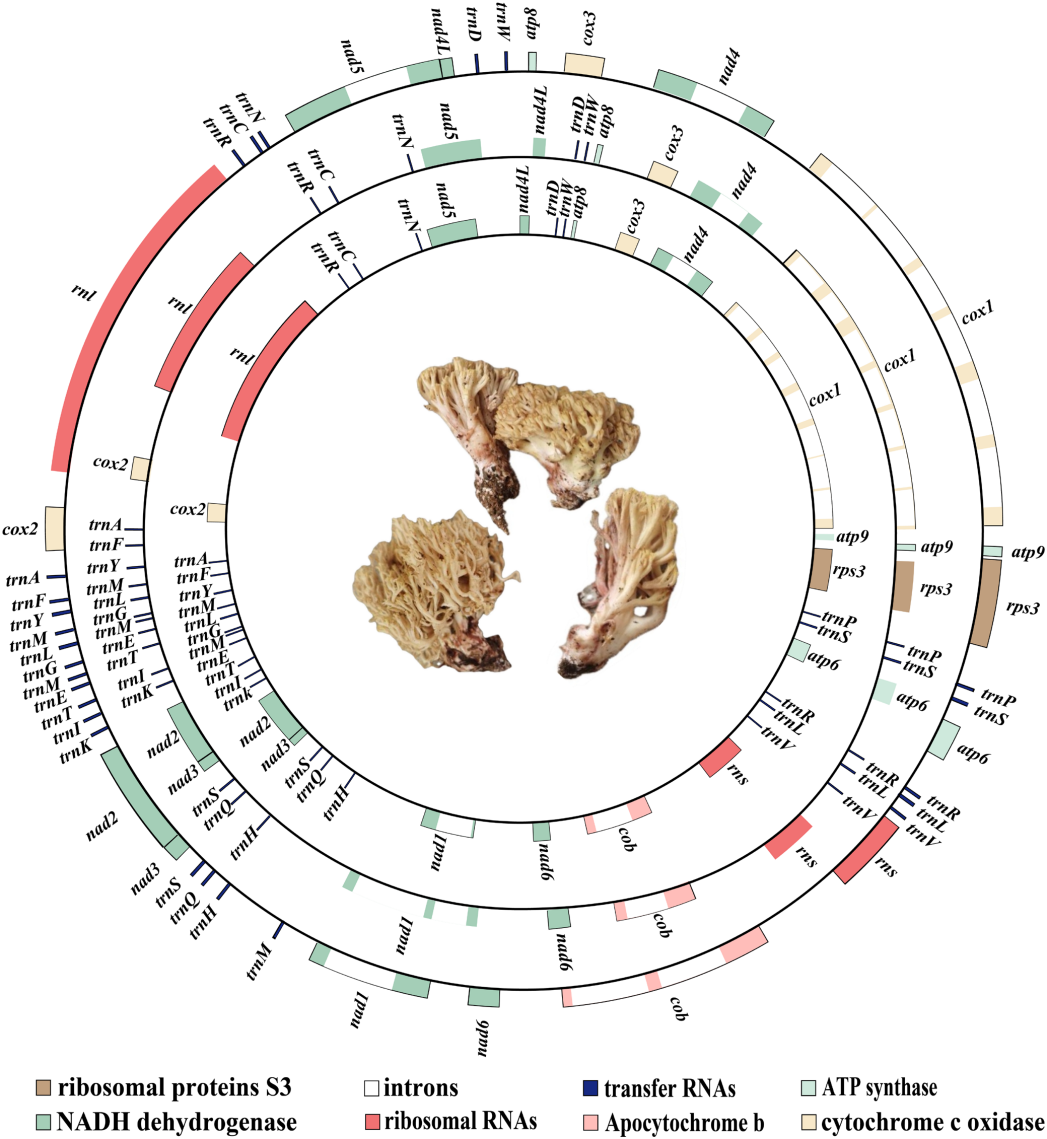
Depictions of the circular organization of the three *Ramaria* mitochondrial genomes. Various genes are illustrated as colored blocks. The concentric rings, moving from the innermost to the outermost, correspond to the mitogenomes of *R. brunnecliacina*, *R. ichnusensis*, and *R. flavescens* .

The complete mitogenomes assembled for *R. brunnecliacina*, *R. ichnusensis*, and *R. flavescens* were found to be 78,960 bp, 61,851 bp, and 81,282 bp in length, respectively (Figure 1). The GC content of these genomes was measured at 24.8%, 27.6%, and 24.7%, respectively (see Additional file 1: Table S1). Analysis of strand asymmetry revealed that all three mitogenomes exhibited negative AT skews and positive GC skews. Additionally, each mitogenome contained a complete set of PCGs, which included *atp6*, *atp8*, *atp9*, *cob*, *cox1*, *cox2*, *cox3*, *nad1*, *nad2*, *nad3*, *nad4*, *nad4L*, *nad5*, *nad6*, and *rps3*.

### 3.2 The RNA Genes Present in the *Ramaria* Mitogenomes

In the *Ramaria* mitochondrial genomes, three ribosomal RNA (rRNA) genes were identified: *rns* and *rnl* (refer to Additional file 1: Table S2), with average lengths of 1667 and 6356 bp, respectively. The genomes also revealed 25 transfer RNA (tRNA) genes, all characterized by the classic cloverleaf structure. Interestingly, there were two distinct tRNA genes for arginine, serine, and leucine, each possessing different anticodons. In contrast, three tRNA genes encoding methionine were found to have identical anticodons. The sizes of the tRNA genes varied between 71 and 88 bp, with *trnS* (tga) and *trnL* (tag) being among the largest due to their extended extra arms. These differences in arm length appear to be the primary factor contributing to the variation in tRNA sizes within the *Ramaria* mitogenomes.

### 3.3 Analysis of Codon Preferences

**FIGURE 2:**
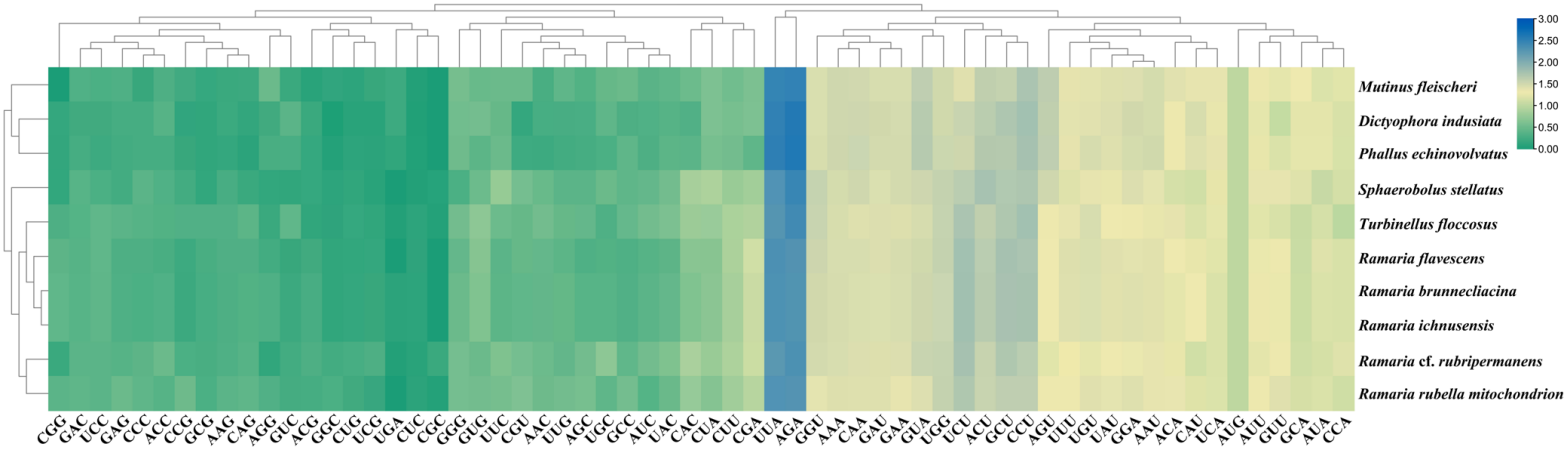
Heatmap showing the Relative Synonymous Codon Usage (RSCU) patterns across the mitogenomes of 10 Phallomycetidae species.

**FIGURE 3:**
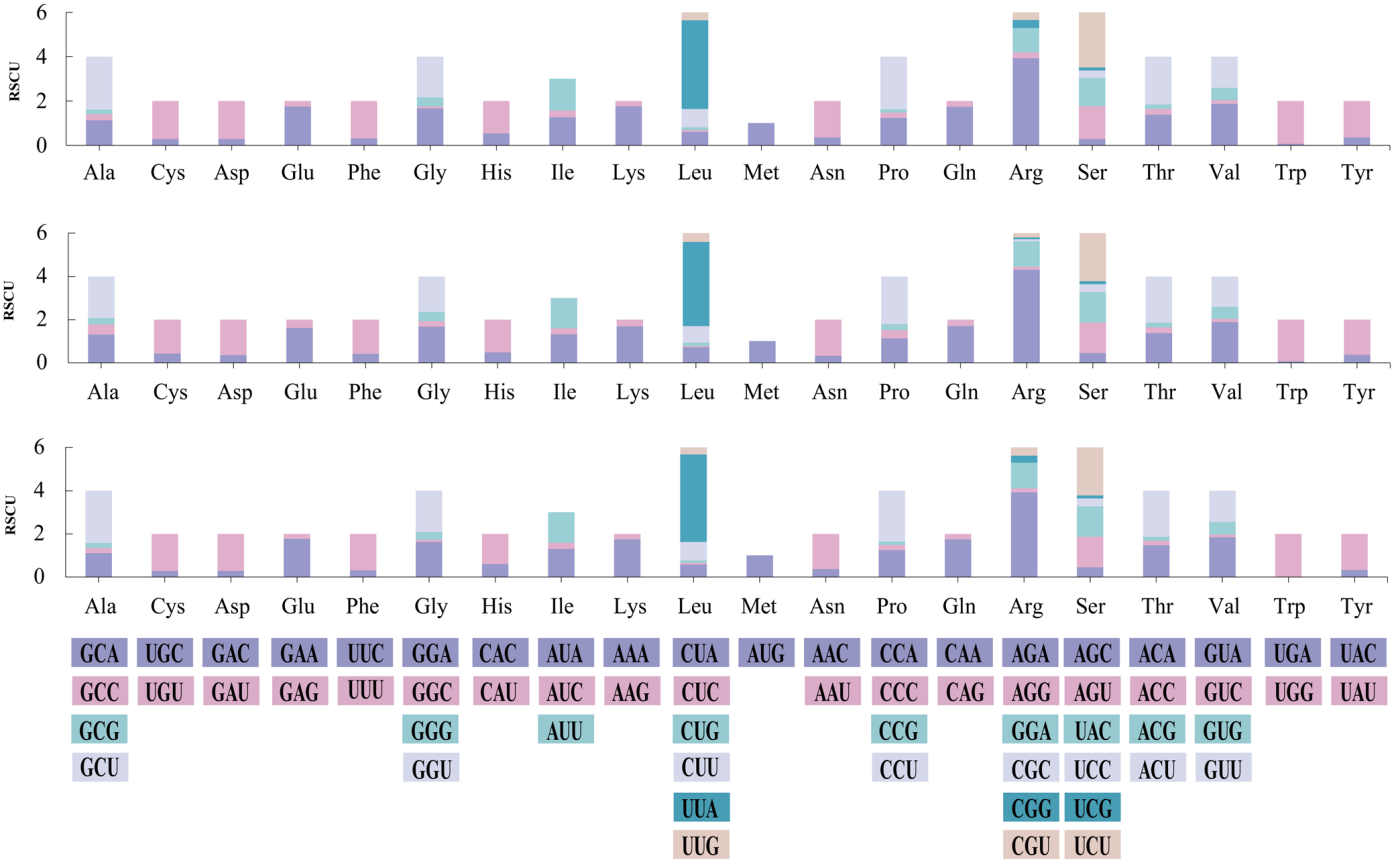
The RSCU in the mitochondrial genomes of three *Ramaria* species, presented as stacked column plots.

Codon use significantly impacts energy expenditure and the pace of protein translation. Mitochondrial genes preferentially utilize specific codons that optimize translation efficiency and contribute to energy conservation for cellular functions. We performed a comparative analysis of codon usage trends in Phallomycetidae mitogenomes by examining 10 mitogenomes—7 from existing databases and 3 newly synthesized *Ramaria* mitogenomes from our present research (Figure 2). Our analysis revealed that the codons AGA (coding for arginine) and UUA (coding for leucine) were the most frequently utilized across the 10 Phallomycetidae species. In particular, the five *Ramaria* species consistently exhibited a codon usage bias for certain amino acids, with UGU predominantly coding for cysteine and GAA for glutamic acid. The mitogenomes of Phallomycetidae species primarily initiated translation with the start codon ATG, whereas the stop codons TAA and TAG were most commonly used. This consistent codon preference observed among *Ramaria* species suggests a genus‐wide pattern. However, significant variations were noted in the start and stop codons across different *Ramaria* species (Figure 3). Additionally, the mitogenomes of *Ramaria* species exhibited a relatively high AT content, averaging 70.14%, reflecting the frequent use of adenine (A) and thymine (T) in their codons.

### 3.4 Evolutionary Rates and Genetic Distances of PCGs

**FIGURE 4:**
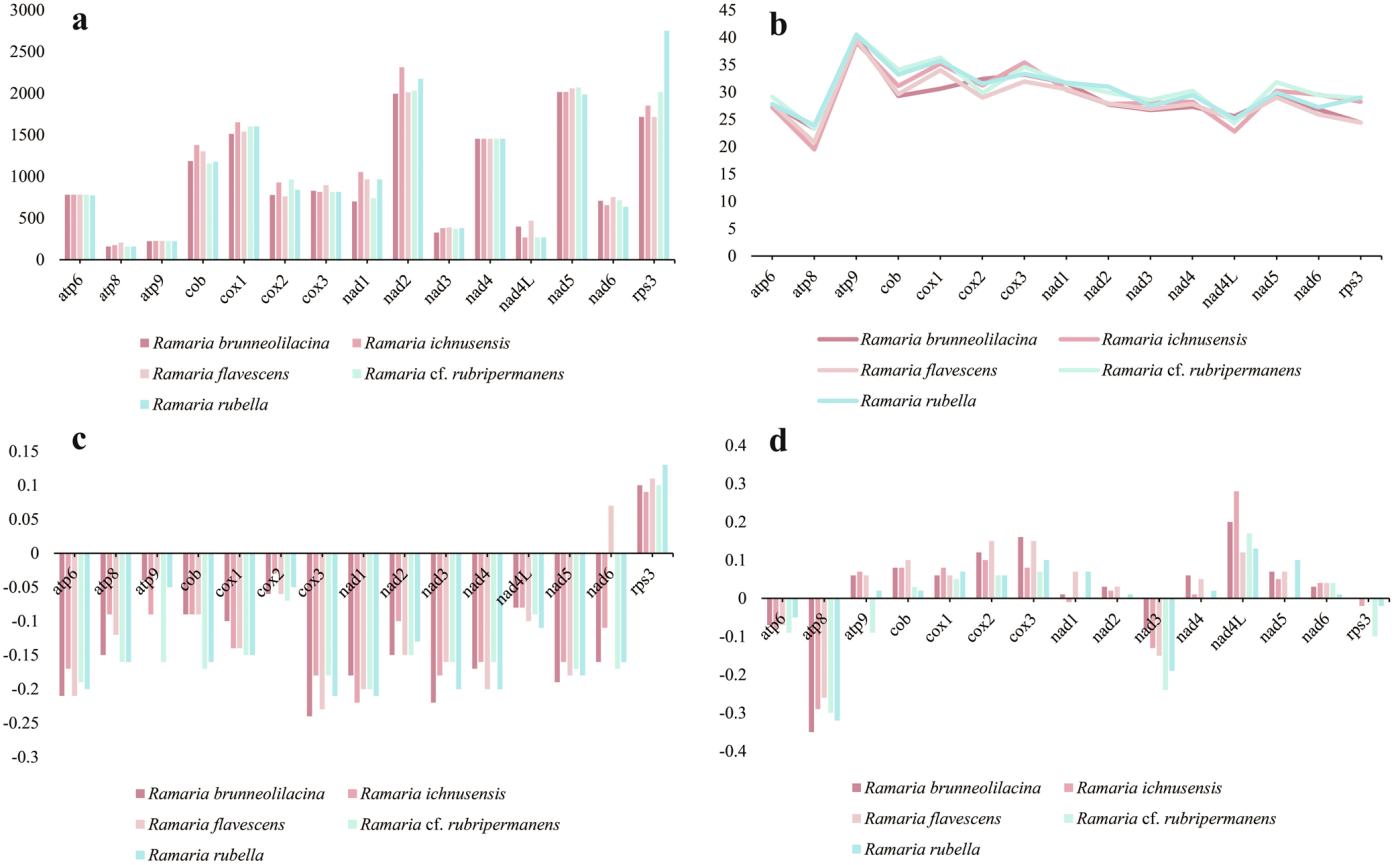
Differences in the length and base composition of 15 protein‐coding genes (PCGs) across five *Ramaria* mitochondrial genomes. (a), Variation in PCG length; (b), GC content of PCGs; (c), AT skew; (d), GC skew.

**FIGURE 5:**
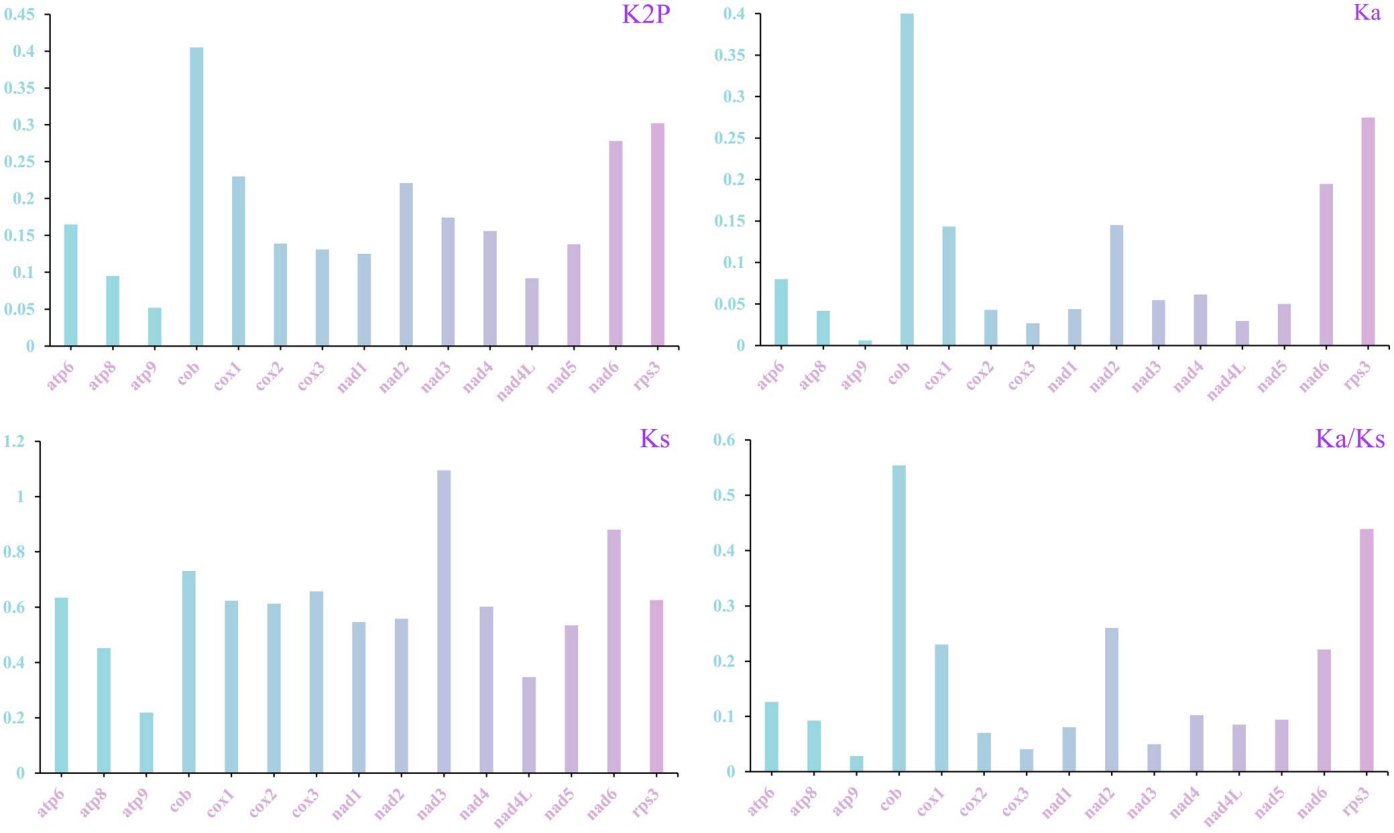
Analysis of the genetic variation in 15 PCGs among five *Ramaria* species. K2P, distance based on the Kimura‐2‐parameter model; Ka, rate of nonsynonymous substitutions per nonsynonymous site; Ks, rate of synonymous substitutions per synonymous site.

Among the 15 PCGs examined across the five *Ramaria* species, *atp6*, *atp9*, and *nad4* were the only ones with consistent gene lengths. The remaining 12 PCGs displayed variations in sequence length (Figure 4). Notably, *rps3* exhibited the greatest length variation, extending up to 1035 bp. Regarding GC content, *atp9* had the highest average value at 39.96%, followed by *cox1* with 34.36%, whereas *atp8* had the lowest average GC content at 22.21%. The varying GC content among these species indicates frequent sequence mutations within the PCGs of *Ramaria*. Among the 15 PCGs, only *rps3* showed a positive AT skew across the five *Ramaria* species, suggesting a general trend toward T‐richness rather than A‐richness on the leading strand of PCGs. GC skew differences among various Phallomycetidae species highlight the prevalence of G/C mutations in these genes. When considering the average Kimura‐2‐parameter (K2P) genetic distance, cob stood out with the highest value among the five *Ramaria* species, closely followed by *rps3*, indicating significant evolutionary divergence in these genes (Figure 5). In contrast, *atp9* had the lowest average K2P genetic distance, suggesting it is highly conserved. *cob* also exhibited the highest nonsynonymous substitution rate (Ka) among the 15 PCGs, with *rps3* and *nad6* following, whereas *atp9* had the lowest Ka. The highest synonymous substitution rate (Ks) was observed in *nad3*, with *atp9* showing the lowest Ks. The Ka/Ks ratios for all 15 PCGs were less than 1, indicating that these genes have been subjected to purifying selection.

### 3.5 Gene Arrangement and Comparative Mitogenomic Analysis

**FIGURE 6:**
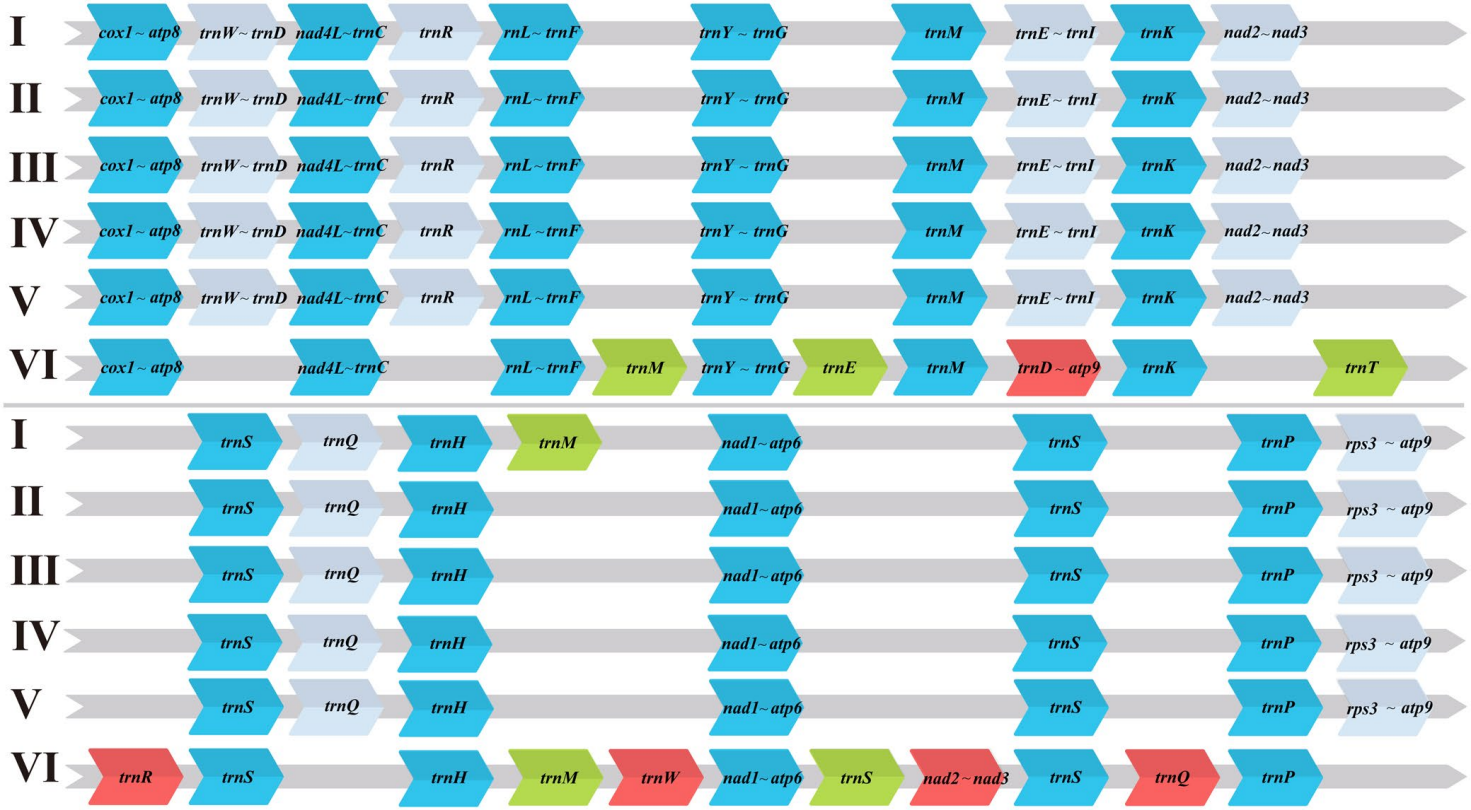
Analysis of mitochondrial gene arrangements across six Phallomycetidae species (with all five *Ramaria* species sharing the same arrangement, denoted as I). Genes highlighted in blue represent identical arrangements across all 10 species; silver blocks indicate genes that share the same arrangement in six out of nine species; red blocks signify genes that have undergone translocation; and green blocks represent genes that the genes have doubled. I: *Ramaria* spices, II: *M*. *fleischeri*, III: *T. floccosus* , IV: *D*. *indusiate*, V: *P*. *echinovolvatus*, VI: *S. stellatus* .

The mitogenomes of the 10 Phallomycetidae species exhibited a conserved arrangement of PCGs. This conservation suggests strong evolutionary pressure to maintain the order of these essential genes, which include components critical for the mitochondrial respiratory chain, such as *cox1*, *cox2*, *cox3*, *nad1‐6*, *atp6*, *atp8*, *atp9*, *cob*, and *rps3* (Figure 6). Similarly, the rRNA genes maintained identical arrangements across the examined *Ramaria* species. The significance of these genes in preserving mitochondrial efficiency and integrity is emphasized by their conserved positioning, which is essential for mitochondrial ribosome function and protein synthesis. The duplication event of *trnM* is a remarkable variation among *Ramaria* species. An additional *trnM* copy may improve the mitochondrial translation machinery, potentially increasing the efficacy of protein synthesis and providing redundancy under specific physiological conditions. The conserved gene arrangements of rRNA genes and PCGs among *Ramaria* species emphasize the significance of these genes in mitochondrial function and indicate that any rearrangements could be detrimental to the organism. The duplication of *trnM* may indicate an adaptive mechanism that improves mitochondrial translation and overall cellular energy production. Gene displacement was detected exclusively in *Sphaerobolus stellatus*, which encompassing *nad2*, *nad3*, *rps3*, and *atp9*. Regarding tRNA genes, *S. stellatus* underwent the relocation of four specific tRNA genes, viz., *trnD*, *trnR*, *trnW*, and *trnQ*. Moreover, the mitogenome of *S. stellatus* has experienced four instances of tRNA duplication involving *trnM*, *trnE*, *trnT*, and *trnS*.

**FIGURE 7:**
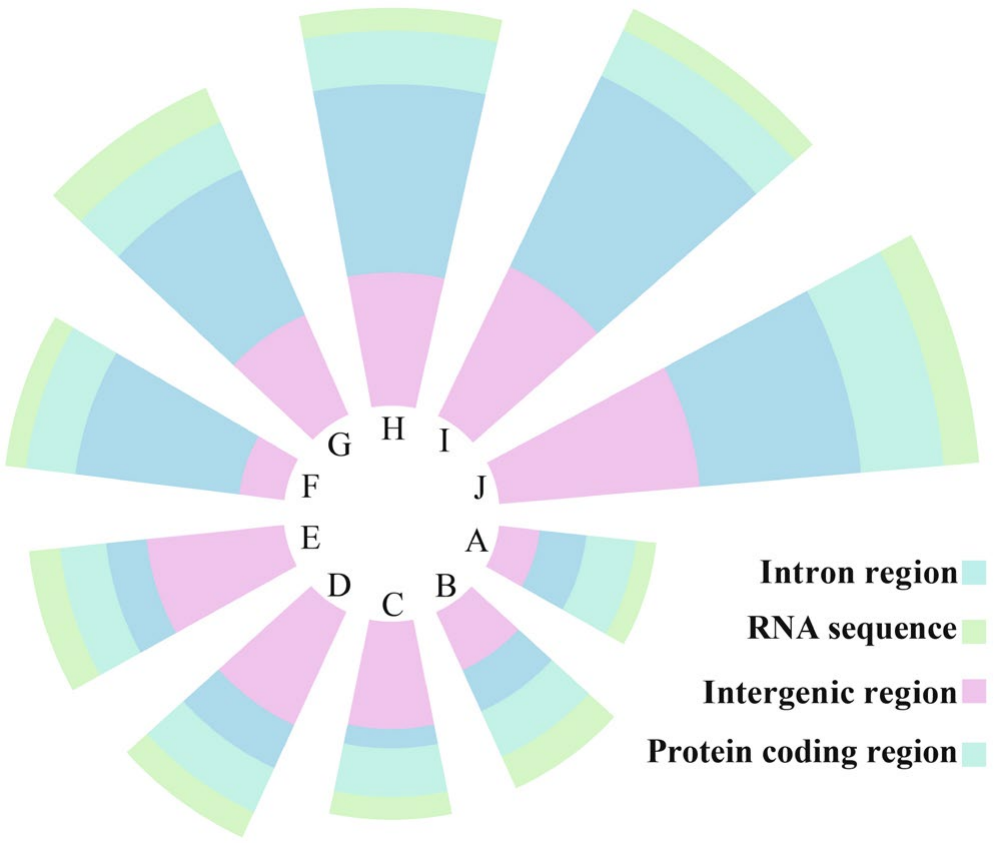
Lengths of RNA region, protein‐coding region, intronic region, and intergenic region in the 10 Phallomycetidae species. A: *P*. *echinovolvatus*, B: *R*. *ichnusensis*, C: *T. floccosus* , D: *R*. *brunnecliacina*, E: *R. flavescens* , F: *D*. *indusiata*, G: *M*. *fleischeri*, H: *R*. cf. *rubripermanens*, I: *R. rubella* , J: *S. stellatus*.

The average size of the 10 Phallomycetidae mitogenomes was 95,960 bp (range, 50,098–152,722 bp) (Figure 7, Additional file 1: Table S1). The mitogenome of *R. ichnusensis* was the second smallest of the 10 Phallomycetidae mitogenomes identified, being larger only than that of *P. echinovolvatus* (50,098 bp) from the order Phallales. The GC content of the six Phallomycetidae mitogenomes varied between 24.3% and 31.69%, with an average GC content of 27.46%. The GC content of the five *Ramaria* mitogenomes was higher than the average. All 10 Phallomycetidae mitogenomes included a pair of rRNA genes. The analysis also revealed the presence of 24–26 tRNA genes in the mitogenomes of the 10 Phallomycetidae species.

### 3.6 Mitogenome Composition Analysis

**FIGURE 8:**
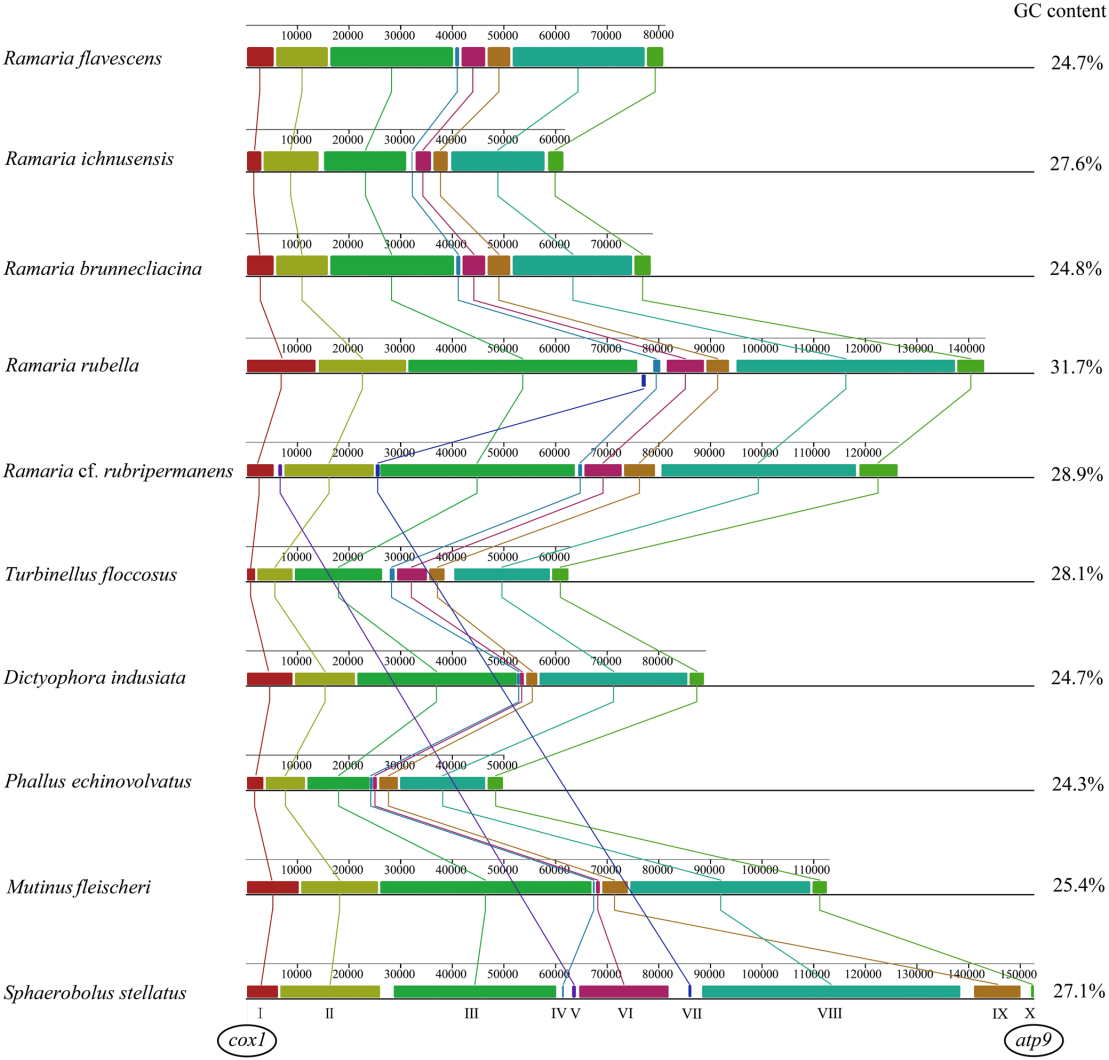
Collinearity analysis map of the 10 Phallomycetidae mitochondrial genomes. Eight homologous regions were detected across the ten mitogenomes. The sizes and positions of homologous regions varied across the mitogenomes.

Comparative genomic analyses of the mitogenomes of the Phallomycetidae species were performed using Mauve, which revealed a multitude of genetic variations and rearrangements (Figure 8). When aligned using progressiveMauve, the mitogenome of Phallomycetidae can be separated into ten homologous sections. The variations in the number and magnitude of these regions among different species reflected the evolutionary and functional diversity within Phallomycetidae. Homologous region IV was limited to *R*. cf. *rubripermanens* and *S. stellatus* , indicating the presence of species‐specific genetic characteristics or adaptations. Homologous region VII was exclusive to *R*. cf. *rubripermanens*, *S. stellatus* , and *R. rubella* , suggesting a distinct evolutionary trajectory or essential physiological role in these particular species. Variations in homologous region I across different species may be associated with the intron of *cox1*. Introns of this nature can potentially affect both gene expression and mitochondrial function. The Phallomycetidae fungus *P*. *echinovolvatus* has the smallest mitochondrial DNA and the least GC content. The decreased size of the genome may be associated with the absence of particular homologous sections and gene content, potentially as an adjustment to specific environmental conditions or metabolic requirements.

### 3.7 Phylogenetic Analysis

**FIGURE 9:**
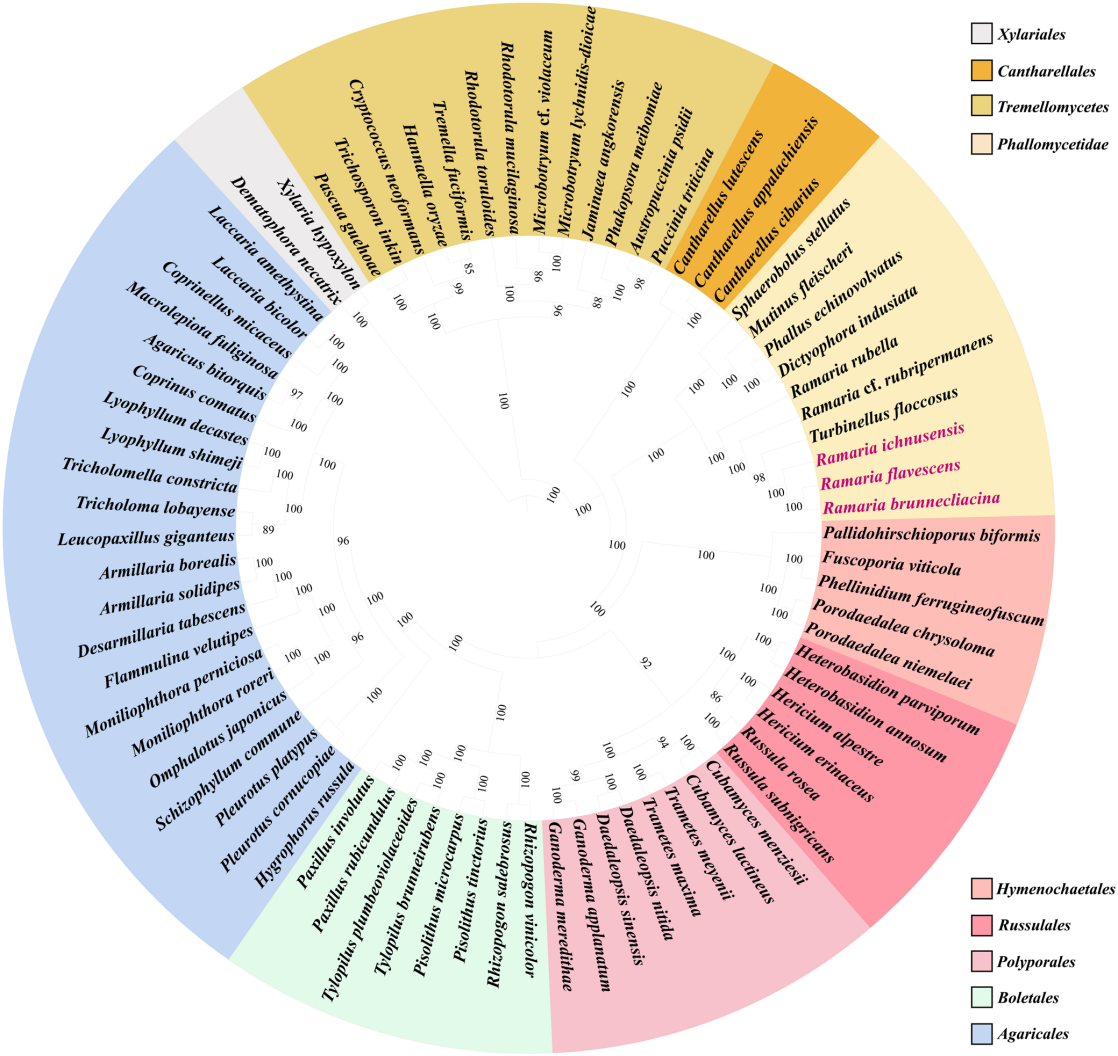
Phylogenetic analysis of 77 Basidiomycota species using maximum likelihood (ML), based on 15 PCGs and 2rRNA sequences.

Morphological characteristics are limited in several Basidiomycota species, and some of these traits frequently overlap, complicating the accurate classification process using morphology alone. In this study, we evaluated the phylogenetic status of 77 Basidiomycota species using a combined mitochondrial gene dataset. A phylogenetic tree that was identical and well‐supported was constructed using both BI and ML methods. Each of the major clades in the study exhibited strong support, with Bayesian posterior probabilities (BPP) of ≥ 0.85 and bootstrap support (BS) values of ≥ 85. The phylogenetic tree categorized the 77 Basidiomycota species (Additional file 1: Table S3) into nine distinct clades (Figure 9). The phylogenetic analysis revealed that *R*. *brunnecliacina* and *R. flavescens* are sister species, indicating a strong and close relationship between them. *T. floccosus* and *Ramaria* formed the same clades, exhibiting a close grouping. The paraphyletic nature of Phallomycetidae was suggested by the fact that the other four species of the subclass formed a distinct branch within the phylogenetic tree. Moreover, these four species exhibited a strong correlation, which contrasts significantly previous studies with the findings of previous studies that were conducted based on morphological identification.

## 4 Discussion

Mitochondrial genomes are essential for the growth and development of eukaryotes, playing key roles in managing oxidative stress and adapting to environmental changes (Cai et al. 2021; Guan et al. 2021). In animals, alterations in mitochondrial DNA can lead to various diseases and influence metabolic rates (Chen et al. 2020). Despite the diversity and complexity of Basidiomycetes (Xu and Wang 2015), detailed studies on their mitochondrial genome structure and variability remain scarce. Currently, public databases contain only 0.05% of the complete mitogenome sequences from all known basidiomycete species. The mitogenomes of Basidiomycetes display considerable diversity in gene content, repetitive DNA sequences, genome organization, and overall size, posing challenges for assembling complete fungal mitogenomes (Basse 2010; Araujo et al. 2021; Mendoza, Perlin, and Schirawski 2020). Within the genus *Ramaria*, significant differences in genome size have been observed. Previous research suggests that fluctuations in fungal mitogenome size are closely linked to plasmid‐derived genes, the accumulation of repeat sequences, dynamic changes in introns, and variations in intergenic regions (Boussau, Brown, and Fujita 2011; Chen et al. 2021; Li et al. 2022; Zubaer, Wai, and Hausner 2018).

Mitochondria originated from an ancient endosymbiotic event in which early eukaryotic ancestors acquired them from bacteria (Archibald 2015; Martin, Garg, and Zimorski 2015; Zimorski et al. 2014). Throughout evolution, the majority of mitochondrial genes were transferred to the nuclear genome, facilitating more integrated cellular regulation (Adams and Palmer 2003; Barton, Jones, and Evolutionary biology. 1977). However, certain PCGs, along with tRNA and rRNA genes, have been retained within the mitochondrial genome, with some PCGs playing crucial roles in the energy metabolism of eukaryotes (Burki 2016). Our study revealed significant variations in the length, codon usage, and base composition of these PCGs among different Phallomycetidae species, including those closely related. The impact of mutations in these PCGs on fungal growth requires an area requiring further investigation.

We also compared the mitochondrial gene arrangement of five *Ramaria* species. The conserved gene arrangements of PCGs and rRNA genes among the *Ramaria* species emphasize the importance of these genes in mitochondrial function and suggest that any rearrangements could be detrimental to the organism. The duplication of *trnM* may reflect an adaptive mechanism to improve mitochondrial translation and overall cellular energy production. We detected the doubling event of tRNAs in five *Ramaria* species. A remarkable variation observed among the *Ramaria* species is the duplication event of *trnM*. An additional *trnM* copy could be advantageous, possibly improving the mitochondrial translation machinery by providing redundancy and potentially increasing the efficiency of protein synthesis under certain physiological conditions. This comparative analysis provides a comprehensive understanding of the mitogenome organization in Phallomycetidae and *Ramaria* species. It emphasizes both the conserved nature of essential mitochondrial genes and the occurrence of specific gene duplication events that may confer functional advantages.

The *Ramaria* genus encompasses a wide variety of fungi, ranging from edible species to highly toxic ones (Barros et al. 2008; Liu et al. 2014). Due to the potential danger of misidentification, which can lead to severe poisoning incidents, accurate classification is critical. However, the close morphological similarities among *Ramaria* species often make it challenging to distinguish them based solely on physical characteristics. In this context, mitochondrial genomes (mitogenomes) offer a robust solution for phylogenetic analysis in eukaryotes (Luchetti and Plazzi 2021). Compared with traditional molecular markers, which require complex processes like multiple PCRs and pyrosequencing, mitogenomes provide a richer source of genetic information with greater convenience. The 15 PCGs and 2 rRNAs genes typically found in mitogenomes serve are reliable markers for establishing evolutionary relationships, offering high support for phylogenetic trees (Nie et al. 2019). This finding is consistent with previous studies based on multiple molecular markers (Giachini et al. 2010), emphasizing the reliability of mitogenomes in analyzing the phylogenetic relationship of Phallomycetidae species. Although nuclear genome‐based analyses can provide more comprehensive genetic data, they are often prohibitively expensive and generate large datasets, limiting their practicality for large‐scale studies in fungi. As a result, mitogenome‐based phylogeny presents a valuable alternative. Our study's phylogenetic analysis demonstrated a closer evolutionary relationship between *R. brunnecliacina* and *R. flavescens* than other *Ramaria* species, aligning with previous research based on multiple molecular markers. This consistency underscores the reliability of mitogenomes in resolving the phylogeny of Phallomycetidae. However, to gain a deeper understanding of the population genetics and evolutionary history of *Ramaria*, it is crucial to expand the available mitogenome data for Phallomycetidae species. Enhancing the mitogenome database will improve our knowledge of genetic diversity within this significant fungal group and provide valuable insights into its evolutionary dynamics.

Several Basidiomycota species exhibit few distinct morphological characteristics, some of which often overlap, making accurate classification based solely on morphology challenging. In our study, the phylogenetic status of 77 Basidiomycota species was evaluated using a combined mitochondrial gene dataset. An identical and well‐supported phylogenetic tree was constructed using both BI and ML methods, with all major clades receiving high support values (BPP ≥ 0.85; BS ≥ 85). According to the phylogenetic tree, the 77 Basidiomycota species were divided into nine major clades, providing a comprehensive overview of their evolutionary relationships. The phylogenetic analysis indicated that *R. brunnecliacina* and *R. flavescens* are sister species, demonstrating a close relationship. This close relationship emphasizes the potential for cryptic speciation within the genus, which cannot be determined based on morphological characteristics alone. *T. floccosus* and *Ramaria* clustered together, forming same clades. In synteny analysis, *R. brunnecliacina* and *R. flavescens* shared a large number of sequence identity regions, and *T. floccosus* and *Ramaria* also shared a large number of highly similar regions. The consistent findings across various analytical methods evidence the reliability of the results. This finding supports the hypothesis that these genera share a common evolutionary ancestor, despite their morphological differences. The other four species of Phallomycetidae formed a separate branch within the phylogenetic tree, indicating that Phallomycetidae is a paraphyletic group. The paraphyly of Phallomycetidae suggests that this group has more complex evolutionary dynamics than that previously reported. Moreover, these four species (*P. echinovolvatus*, *D. indusiate*, *M. fleischeri*, and *S. stellatus* ) demonstrated a close relationship that differed significantly from previous results based on morphological identification. Four trees together support the topology of ((( *R. flavescens* + *R*. *brunneolilacina*) + *R*. *ichnusensis* + *T. floccosus* + *R*. cf. *rubripermanens* + *R. rubella* )) + ((*D*. *indusiata* + *P*. *echinovolvatus*) + *M*. *fleischeri* + *S. stellatus* ))) (Figure S1). This discrepancy emphasizes the importance of molecular data in resolving phylogenetic relationships, particularly in groups wherein morphological convergence or plasticity is prevalent (Martín et al. 2021). Molecular phylogenetics can provide more accurate insights into evolutionary relationships and species boundaries, which are important for understanding the biodiversity and evolutionary history of Basidiomycota. Overall, this study emphasizes the limitations of morphological classification in Basidiomycota and demonstrates the utility of mitochondrial gene datasets in constructing robust phylogenetic trees. These findings contribute to a deeper understanding of the evolutionary relationships within Basidiomycota, providing a more detailed framework for future taxonomic and ecological investigations. The high support values for the major clades also suggest the reliability of the mitochondrial genes used in this study for phylogenetic analysis in Basidiomycota.

## 5 Conclusions

We have successfully sequenced and characterized three novel mitochondrial genomes from the *Ramaria* genus, namely *R. brunnecliacina*, *R. ichnusensis*, and *R. flavescens* . These genomes revealed significant differences in genetic content, gene lengths, tRNA compositions, and codon usage patterns. Our comparative genomic analysis identified substantial gene rearrangements within Phallomycetidae mitogenomes, including gene translocations and tRNA duplications. Phylogenetic analysis showed that *R. brunnecliacina* and *R. flavescens* share a closer evolutionary relationship compared with other *Ramaria* species. This finding is consistent with previous phylogenetic studies using multiple molecular markers, underscoring the value of mitochondrial genomes in resolving phylogenetic relationships within Phallomycetidae. This research provides a critical foundation for future studies on the evolution, genetics, and taxonomy of this significant genus and its related fungal groups. Deepening our understanding of *Ramaria* mitogenomes will offer greater insights into the evolutionary relationships and genetic diversity within this fascinating group of fungi.

## Author Contributions

**Hongmei Liu:** conceptualization (equal), funding acquisition (equal), project administration (equal), resources (equal), supervision (equal). **Yaohang Long:** data curation (equal), formal analysis (equal), methodology (equal), writing – review and editing (equal). **Yaping Wang:** data curation (equal), formal analysis (equal), funding acquisition (equal), investigation (equal). **Gongyou Zhang:** conceptualization (equal), data curation (equal), investigation (equal). **Zhongyao Guo:** conceptualization (equal), data curation (equal), methodology (equal), visualization (equal), writing – original draft (equal). **Xianyi Wang:** conceptualization (equal), data curation (equal), investigation (equal), methodology (equal), writing – original draft (equal). **Guoyu Wang:** data curation (equal), formal analysis (equal), investigation (equal). **Jiawei Tao:** data curation (equal), formal analysis (equal), visualization (equal).

## Conflicts of Interest

The authors declare no conflicts of interest.

## Supporting information


Appendix S1.

Appendix S2.

## Data Availability

The complete mitogenomes of *R*. *brunnecliacina*, *R*. *ichnusensis*, and *R. flavescens* were deposited in the GenBank database (Benson et al. 2018) under the accession numbers PP847336, PP847337, and PP847338, respectively.
