# Supplementary material for: Phylogenetic Relationships of Three Ramaria Species Based on Mitochondrial Genome Analysis
Source: Ecol Evol. 2025 Feb 12;15(2):e70901. doi: 10.1002/ece3.70901 (PMC11815223; doi:10.1002/ece3.70901)
Supplement: Supplementary file 1 — Appendix S1. [file ECE3-15-e70901-s002.pdf]

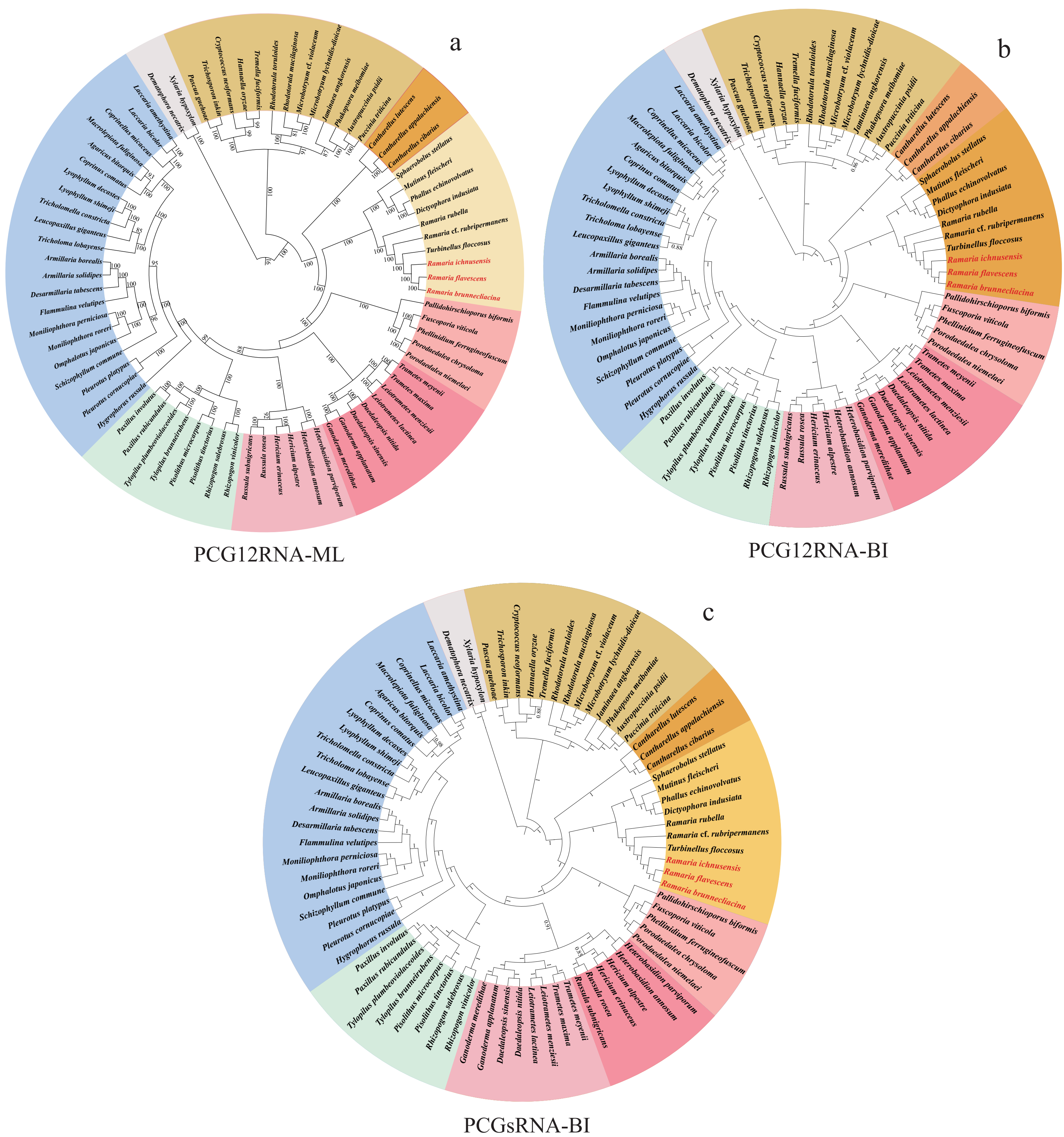

a: Phylogenetic analysis of 77 Basidiomycota species using maximum likelihood (ML), based on comprising the first and second codon positions of these 15 PCGs and 2rRNA sequences.
b: Phylogenetic analysis of 77 Basidiomycota species using Bayesian inference (BI), based on comprising the first and second codon positions of these 15 PCGs and 2rRNA sequences.
c: Phylogenetic analysis of 77 Basidiomycota species using Bayesian inference (BI), based on 15 PCGs and 2rRNA sequences.
